# Roles of the Rlim-Rex1 axis during X chromosome inactivation in mice

**DOI:** 10.1073/pnas.2313200120

**Published:** 2023-12-19

**Authors:** Feng Wang, Ashmita Chander, Yeonsoo Yoon, Janelle M. Welton, Mary C. Wallingford, Carmen Espejo-Serrano, Francisco Bustos, Greg M. Findlay, Jesse Mager, Ingolf Bach

**Affiliations:** 1 Department of Molecular, Cell and Cancer Biology, University of Massachusetts Chan Medical School, Worcester, MA 01605, USA; 2 Department of Veterinary & Animal Sciences, University of Massachusetts Amherst, Amherst, MA 01003, USA; 3 Division of Genes and Development, Department of Pediatrics, University of Massachusetts Chan Medical School, Worcester, MA 01605, USA; 4 MRC Protein Phosphorylation & Ubiquitylation Unit, School of Life Sciences, University of Dundee, Dundee DD1 5EH, UK

## Abstract

In female mice the gene dosage from X chromosomes is adjusted by a process called X chromosome inactivation (XCI) that occurs in two steps. An imprinted form of XCI (iXCI) that silences the paternally inherited X chromosome (Xp) is initiated at the 2-4 cell stages. As extraembryonic cells including trophoblasts keep the Xp silenced, epiblast cells that give rise to the embryo proper reactivate the Xp and undergo a random form of XCI (rXCI) around implantation. Both iXCI and rXCI require the lncRNA *Xist*, which is expressed from the X to be inactivated. The X-linked E3 ubiquitin ligase Rlim (Rnf12) in conjunction with its target protein Rex1 (Zfp42), a critical repressor of *Xist*, have emerged as major regulators of iXCI. However, their roles in rXCI remain controversial. Investigating early mouse development, we show that the Rlim-Rex1 axis is active in pre-implantation embryos. Upon implantation Rex1 levels are downregulated independently of Rlim specifically in epiblast cells. These results provide a conceptual framework of how the functional dynamics between Rlim and Rex1 ensures regulation of iXCI but not rXCI in female mice.

## Introduction

Eutherian female mammals adjust the gene dosage from X chromosomes between sexes by a process known as X chromosome inactivation (XCI), a paradigm for the study of epigenetic gene silencing. In mice, XCI occurs in two waves. Beginning at the late two cell-/early four cell stage, imprinted XCI (iXCI) exclusively silences the paternal X (Xp). While extraembryonic cell types including trophoblasts maintain this XCI pattern, cells in the inner cell mass (ICM) specifically reactivate the Xp (XCR) and undergo a random form of XCI (rXCI) around implantation, where each cell inactivates either the maternal X (Xm) or the Xp (1, 2). The long non-coding RNA (lncRNA) *Xist* represents a key regulator of both rXCI and iXCI. *Xist* RNA paints the inactive X from which it is expressed, thereby triggering downstream repressive chromatin modifications including H3K27me3 that lead to X-silencing (3).

In mice, the X-linked *Rlim* (Rnf12) gene encodes a RING finger E3 ligase that is crucial for iXCI (4, 5). Female pre-implantation embryos lacking *Rlim* (KO) display severe inhibition of *Xist* transcription and defective X dosage compensation, and die shortly after implantation due to defective placental trophoblast development (5, 6). Rlim exerts its activity to induce *Xist* by proteasomal targeting of Rex1 (reduced expression gene 1)(7), a gene expressed in undifferentiated embryonic stem cells (8, 9). There, Rex1 functions as a transcriptional repressor of *Xist* (7), and in female mice the *Rlim* KO phenotype including iXCI is rescued in *Rlim/Rex1* double KO animals (10), illustrating the critical function of the Rlim-Rex1 axis in controlling iXCI.

During pre-implantation development Cdx2-induced trophectodermal cells (TEs) form the first epithelium as outside layer of the embryo (11). At the blastocyst stage, TEs are segregated into mural and polar TE (mTE; pTE) with pTE cells located in close proximity to the ICM. Upon implantation mTE cells quickly differentiate into trophoblast giant cells (TGCs) that form the parietal yolk sac. In contrast, pTE cells contribute to most placental trophoblast cell types and give rise to the extraembryonic ectoderm (exe) structure that harbors and maintains this progenitor cell type. The pTE progenitor state requires Fgf4 (12–14), which is synthesized by epiblast cells, and its receptor Fgfr2c (15) through mitogen-activated protein kinase (MAPK) signaling via Raf/Mek/Erk (16–18). This pathway activates a transcription factor network that includes Cdx2, Esrrb, Eomes and Elf5 among others (19–24). Trophoblast development requires X dosage compensation, as deletion of *Xist* in female embryos results in loss of pTEs (25). However, the involved mechanisms leading to this phenotype remain unclear. Moreover, despite major functions in female pre-implantation embryos, the developmental dynamics of Rlim-Rex1 expression and the precise defects caused by axis disturbance are unknown. This issue is particularly important as evidence in mice indicates that *Rlim* is dispensable for rXCI in mice (26), but current models require doses of Rlim transcribed from two X chromosomes for activating *Xist* during rXCI (1). Thus, as these ESC-derived models appear incompatible with the *in vivo* data, the roles of the Rlim-Rex1 axis in epiblast cells remain unclear.

We show that lack of X dosage compensation in female mouse embryos causes cell fate confusion specifically of pTE progenitors, leading to pre-gastrulation lethality. Comparing the dynamic expression of Rlim and Rex 1, we find an inverse correlation of both proteins during pre-implantation development, consistent with crucial functions during iXCI. However, this functional connection is severed upon implantation specifically in the epiblast cell lineage, allowing for Rlim-independent rXCI. Our data provide a developmental justification for a strict requirement of X dosage compensation early during mouse embryogenesis and resolve underlying mechanisms of how iXCI but not rXCI is selectively regulated by a dynamic interplay between Rlim and Rex1.

## Results

### Rlim activity is required specifically in pTE precursor cells in the exe

We first examined the precise and sex-specific developmental consequences of disrupting the *Rlim* gene with an emphasis on trophoblast cell types. To efficiently generate embryos lacking *Rlim*, we crossed females carrying a maternal Sox-2-Cre mediated *Rlim* cKO and a paternal *Rlim* KO allele (*Rlim* flox_m_/KO_p_; Sox2-Cre^+/-^) with males lacking *Rlim* (KO/Y; Fig. S1A). This mating strategy ensures that all offspring lack *Rlim* (6, 26). As our previous studies suggested that lack of *Rlim* has no major effect on pre-implantation development (5), we started out by interrogating female blastocyst outgrowths, comparing them to males that do not undergo XCI and served as controls. Blastocysts were isolated at E3.5 and cultured up to 5 days. The sex was determined via PCR, after image recording. At day 1 of culturing, mTEs stained Cdx2^+^ and no obvious differences were observed between female and male embryos (Fig. S1B). At 3 days in culture both male and female embryos had hatched, with similar numbers of mTE-derived differentiated giant cells, as previously reported (5). Moreover, overall rates of mitosis and cell death at this stage appeared not significantly different between sexes as judged by staining with antibodies against proliferation marker phospho-histone 3 (pH3) (Fig. S1B) or apoptosis marker cleaved Caspase 3 (26). However, Cdx2 was detectable but with notably decreased signal in female embryos. At day 5, all male outgrowths (n=14) but none of the females (n=12) displayed an outgrowth likely consisting of pTE-derived cells (Fig. S1B, C). These results are consistent with published results (5, 26) and suggest that major developmental effects caused by lack of Rlim are in pTE-derived cell types, specifically in females.

**Figure 1:**
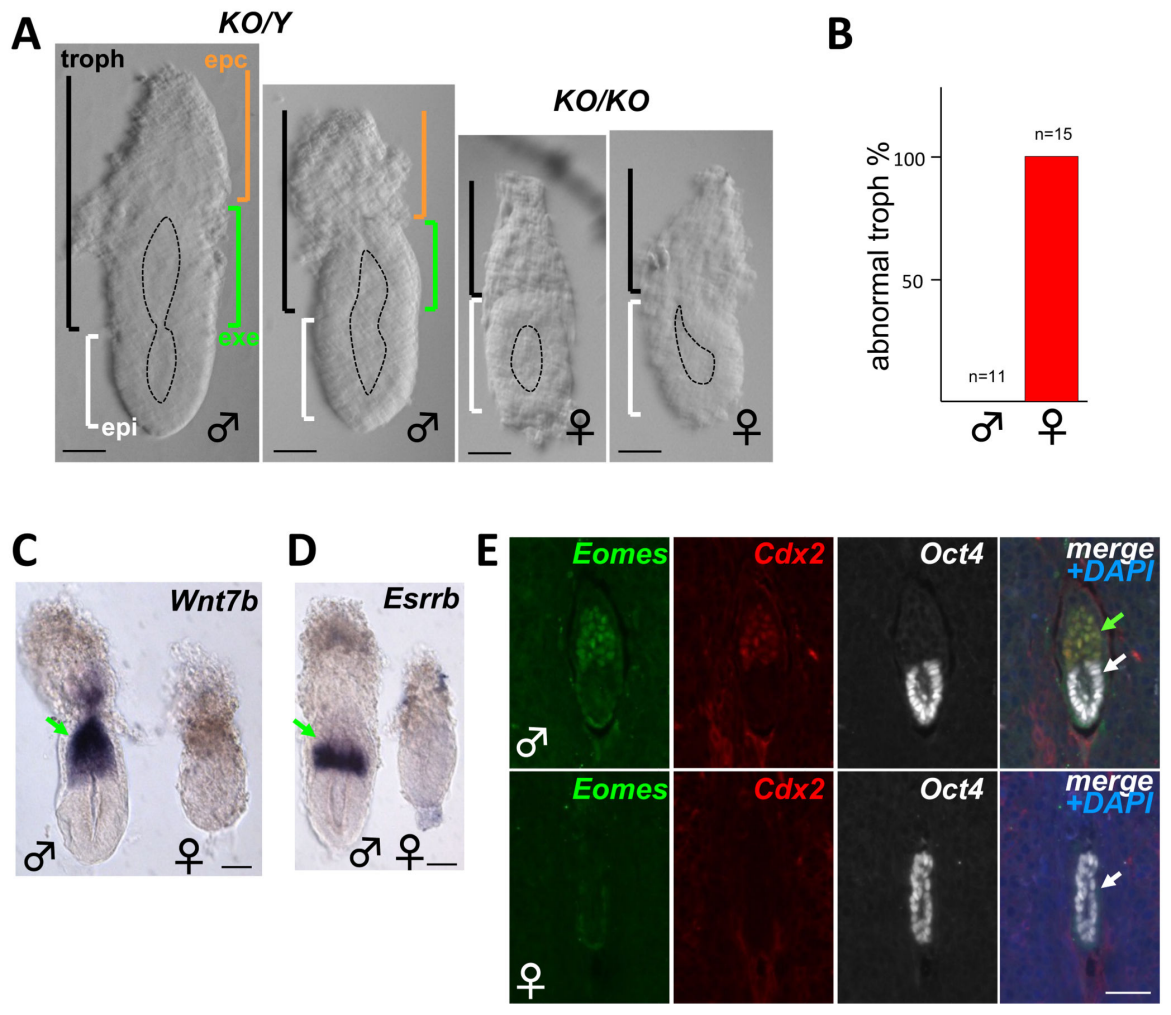
Absence of the exe structure in female embryos lacking *Rlim*. Male/female littermates within subfigures are shown at the same magnification. Sex was determined after image recording. **A)** Representative brightfield images of E5.75 embryos. Approximate embryo and lumen domains are indicated. n>27 each. epc=ectoplacental cone region (brown); exe=extraembryonic ectoderm region (green); troph= trophoblast-derived (black); epi=epiblast (white). Scale bars = 60 μm. **B)** Quantification of phenotypic appearance. **C, D)** Whole embryo *in situ* hybridization. Lack of exe markers Wnt7b (C) and Esrrb (D) in E5.75 *Rlim* KO females (n>10, each). Scale bars = 60 μm. **E)** IHC of *Rlim* KO embryonic sections at E5.5 within decidua reveals lack of exe markers Eomes and Cdx2. Genotyping after image recording. Scale bar = 40 μm. In C-E green and white arrows indicate exe and epiblast regions, respectively.

To visualize embryonic structures including the epiblast (epi), exe and ectoplacental cone (epc), we continued our analyses on dissected early post-implantation embryos at developmental stages E5.5 and E6.5. While epiblast tissues, epiblast lumenogenesis and mTE-derived parietal yolk sac were present both in male and female embryos, presumed pTE-derived trophoblast domains appeared disorganized in 100% of females examined (Fig. 1 A, B). Using wholemount *in situ* hybridization or IF on sections of embryos within decidua failed to detect exe markers including Wnt7b (27, 28), Esrrb (22, 29), Eomes (24) and Cdx2 (11, 30) (Figs. 1C-E). The lack of these markers that are normally expressed in specific exe subdomains suggest a complete absence of pTE-derived cell types that make up the exe. In contrast, Oct4^+^ (31, 32) epiblast tissue was present (Fig. 1E), indicating exe-specific defects in *Rlim* null female embryos. These results are fully consistent with data obtained in female embryos with a paternally inherited mutated *Xist* allele (25).

### Lack of Rlim causes cell fate confusion of pTE precursor cells

**Figure 2:**
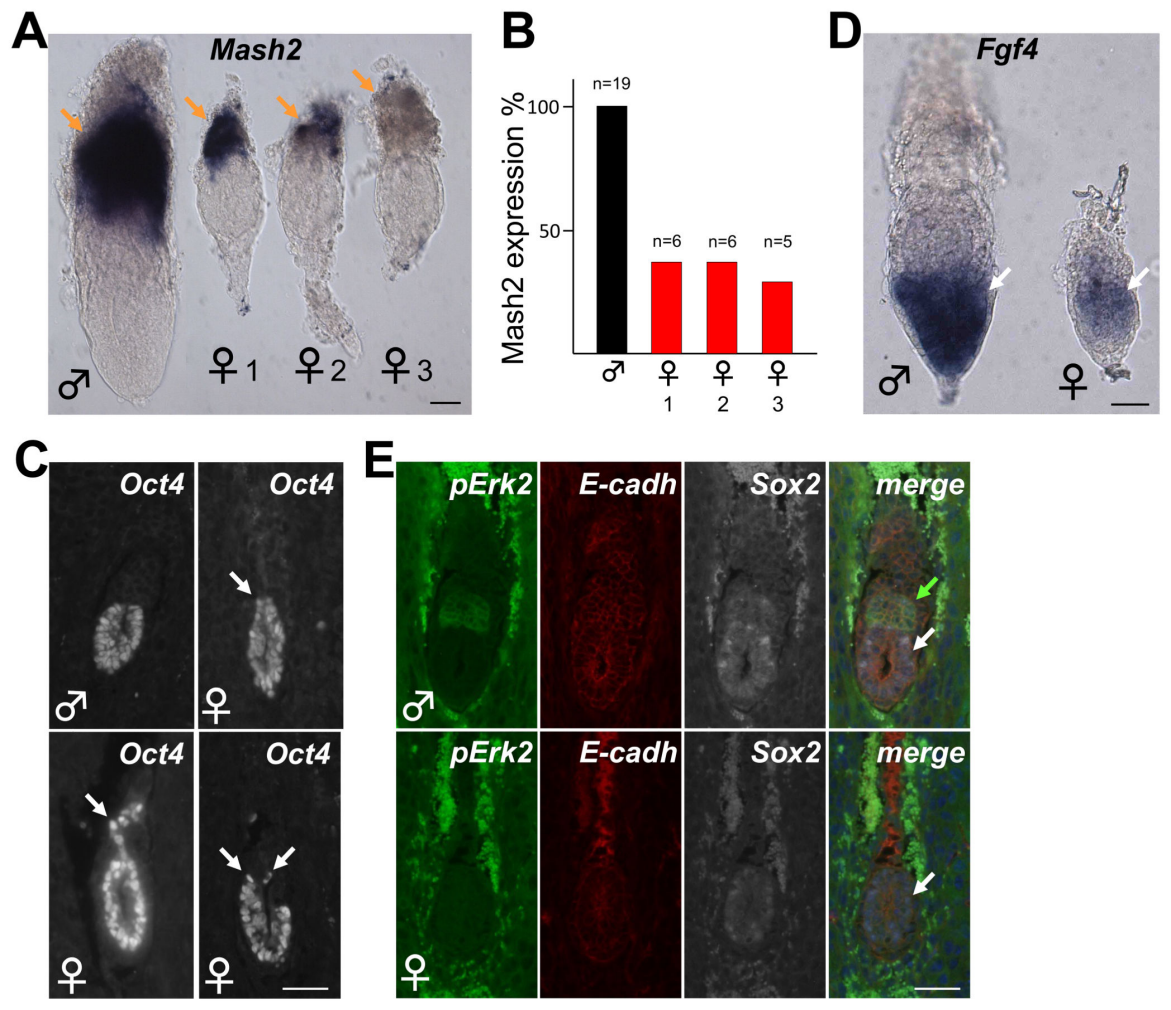
Lack of iXCI causes pTE cell fate confusion. Representative images of male and female *Rlim* KO littermate embryos at E6.5. A, C: whole mount ISH; B, D: IHC, sections. Genotyping after image recording. **A)** *In situ* hybridization with epc marker Mash2 reveals female embryos with strong (1), intermediate (2) and weak/no staining (3). Scale bar = 50μm. **B)** Summary of A. **C)** Examples of undefined epi/troph border and/or expansion of Oct4^+^ cells into trophoblast domains (arrows). Scale bar = 40μm. **D)** *In situ* hybridization of embryos at E6.5 using Fgf4 antisense probe. Note expression of Fgf4 in the epiblast of female *Rlim* KO embryos. Scale bar = 50μm. **E)** Absence of pErk2^+^ cells in *Rlim* KO females. Same magnification of images within sub-figures. Scale bar = 40μm. Brown arrows = epc or epc-like; white arrows = epiblast or epiblast-like; green arrow = exe.

Even though the exe structure is entirely absent in *Rlim* KO females, pTE-derived cells appear present albeit at low quantity (Fig. 1A). Examining these tissues, we tested expression of Mash2 (29, 33) a marker for spongiotrophoblast cell types in the epc using whole embryo *in situ* hybridization. Interestingly, while some female embryos exhibited strong staining comprising the entire trophoblast domain, partial/intermediate or no/weak Mash2 staining was detected in others (Fig. 2A), and these Mash2 expression patterns appeared in similar frequencies (Fig. 2B). We also noted that many female embryos lacking *Rlim* did not develop a defined epiblast-trophoblast boundary and that Oct4^+^ cells were detectable well into the trophoblast domain (Figs. 1E; 2C). Because Fgf4 expressed by epiblast cells is crucial for the maintenance of pTE progenitor cells (14), we tested Fgf4 expression in female embryos via whole embryo in situ hybridization. Indeed, while the Fgf4 signal in males was more robust, female embryos at E6.5 still expressed Fgf4 mRNA, even though cells in the trophoblast compartment expressing activated phospho-Erk1,2 (p-Erk) were no longer detectable (Figs. 2D, E). Thus, loss of pTE progenitors occurs in the presence of Fgf4, indicating a cell-autonomous requirement of X dosage compensation by this cell type. Combined with published results, these data suggest that lack of Rlim inhibits iXCI (5, 6), leading to pTE progenitor cell fate confusion and premature differentiation into various cell types including the Mash2^+^ spongiotrophoblast lineage.

### Inverse correlation of Rlim and Rex1 protein expression in pre-implantation embryos

Next, we investigated the underlying mechanisms for this phenotype and the roles of the Rlim-Rex1 axis by examining the expression profiles of both proteins during pre-implantation development. To obtain high quality antibodies, we developed a polyclonal Rex1 antibody raised in sheep recognizing aa 1-288 of mouse Rex1 (34). Indeed, when tested on male embryonic stem cell (ESC) models by Western blot or by immunostaining, the Rex1 antiserum recognized Rex1 in nuclei of WT ESCs and ESCs lacking *Rlim*, but not ESCs carrying an additional Rex1 deletion (Figs. S2A, B, respectively). The levels of Rex1 detected in single *Rlim* KO ESCs were increased when compared to WT cells, consistent with Rlim’s E3 ligase activity regulating Rex1 levels (7). These results reveal not only a high specificity of the Rex1 antiserum but also illustrate that the Rlim-Rex1 axis is active in male ESCs.

**Figure 3:**
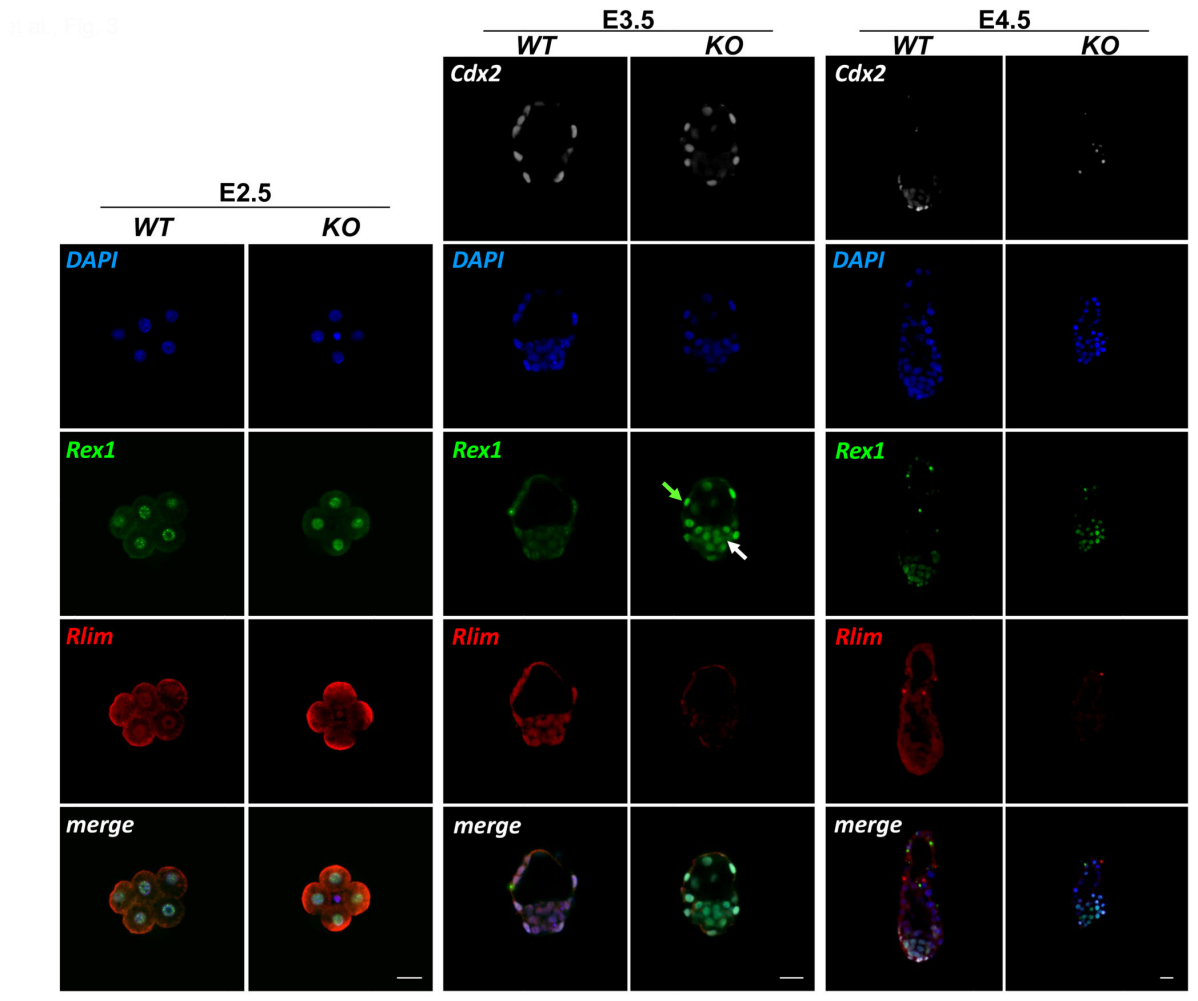
Increased Rex1 levels in pre-implantation embryos lacking Rlim. WT and *Rlim* KO embryos at stages E2.5, E3.5 and E4.5 were co-stained in parallel using indicated antibodies. Image recordings/processing of KO and control embryos were carried out with the same settings. Rlim=red; Rex1=green; Cdx2=white. DAPI=blue. Green and white arrows indicate trophoblast and epiblast regions, respectively. Note elevated Rex1 immunoreactivity in embryos lacking *Rlim* in both cell types. Scale bars=25 μm.

To illuminate the dynamics of Rlim and Rex1 expression during iXCI *in vivo*, we performed co-immunostaining on whole embryos, using this Rex1 antibody in conjunction with a previously described rabbit antibody recognizing the N-terminal portion of Rlim (35), comparing animals lacking *Rlim* (Fig. S1A) with WT controls. We first focused our analyses on whole pre-implantation embryos stages E2.5, E3.5 and E4.5. Because *Rlim* is among X-linked genes that are quickly silenced during the iXCI process, it is mostly expressed from the maternal allele at these stages in both sexes (36), and thus, *Rlim* effects on Rex1 levels in male and female embryos are comparable. As previously reported (5, 26), in WT embryos Rlim is expressed throughout pre-implantation development (Fig. 3). Likewise, Rex1 expression was low but detectable in these embryos at all stages (Fig. 3), with strictly nuclear localization. At E2.5 Rex1 protein was also detected in specific nuclear punctae which, as judged by comparison with DAPI staining, did not correspond to centric heterochromatin domains. Rlim localization in cells was somewhat more diffuse indicating nuclear and some cytoplasmic localization, suggesting shuttling between these compartments (37), co-localizing with Rex1 in the nucleus. Starting at E4.5, general Rlim immunoreactivity specifically in the ICM harboring epiblast cells appeared lower (Fig. 3), when compared to trophoblasts as previously reported (26). Because we detected elevated Rex1 immunoreactivity in embryos lacking *Rlim* throughout pre-implantation development that appeared to peak at E3.5 (Fig. 3), we focused further analyses at this stage. Evaluating a published single embryo RNA-seq dataset (6) revealed similar Zfp42 (Rex1) mRNA levels in male and female E3.5 embryos with or without Rlim (Fig. S3A). However, quantification of IHC Rex1 signals via ImageJ revealed that low levels of Rex1 protein are Rlim-dependent both in cells of the ICM and trophoblasts irrespective of sex (Figs. S3B, C). The Rlim-Rex1 protein expression profiles at pre-implantation stages are consistent with a gradual upregulation of Rex1 mRNA from low levels at the 4 cell stage to high levels at blastocyst stages (38), and indicate that the Rlim-Rex1 axis is active throughout pre-implantation development in most/all cells of the embryo.

### Rlim-independent downregulation of Rex1 specifically in epiblast cells of implanting embryos

**Figure 4:**
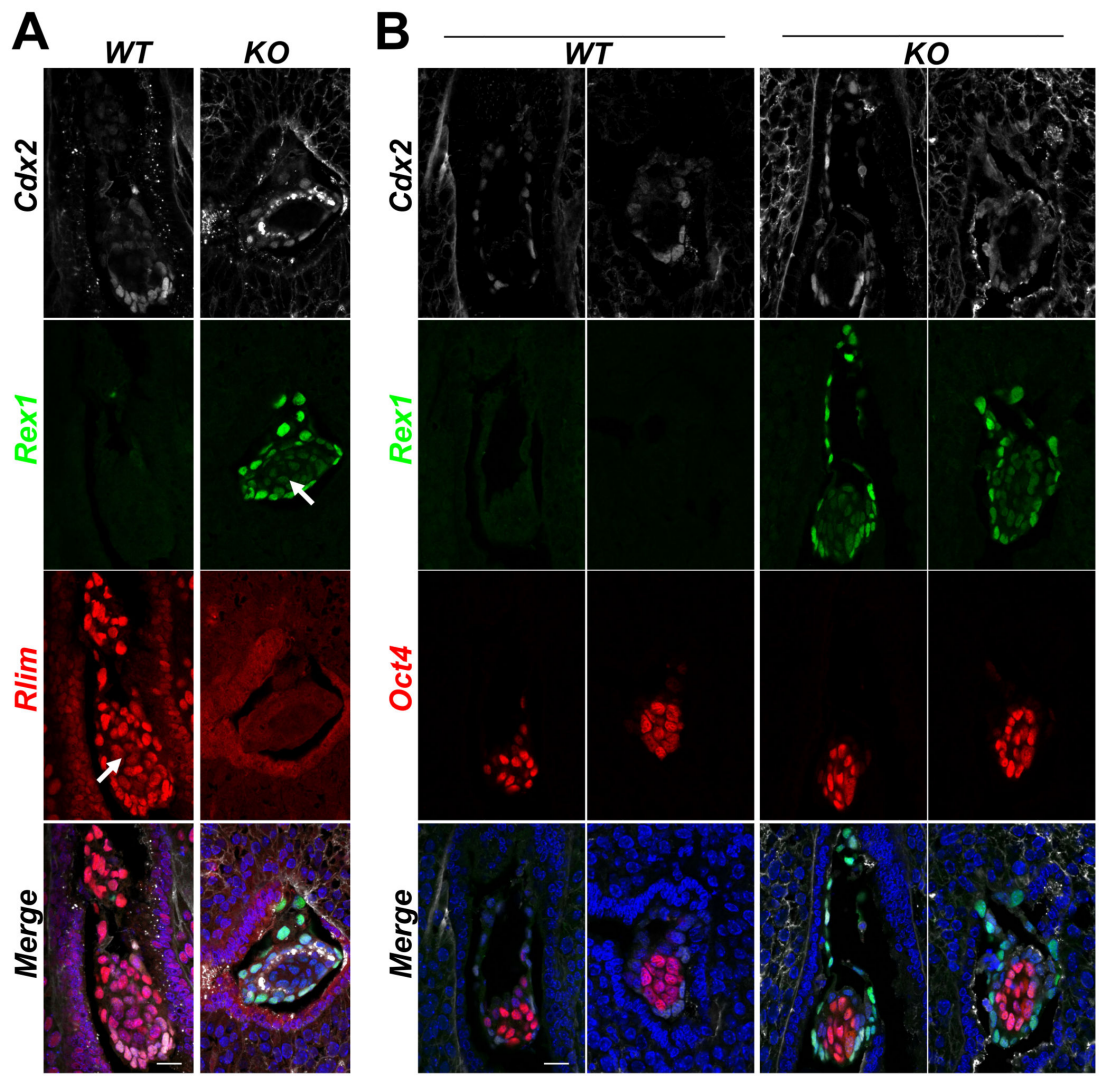
Rlim-independent downregulation of Rex1 in epiblast cells upon implantation. Sections of WT and *Rlim* KO embryos in uteri at post-implantation stage E5.0 were co-stained in parallel using indicated antibodies. Image recordings/processing of KO and control embryos were carried out using the same settings. **A)** Co-staining Rlim and Rex1. **B)** Co-staining Oct4 and Rex1. White arrows indicate epiblast domains. Note decreased Rex1 immunoreactivity specifically in Oct4^+^ epiblast regions in embryos lacking *Rlim*. Scale bars =50 μm.

The role of Rlim in the initiation of Xist during rXCI is controversial. Based on data from ESCs, the current model of triggering rXCI requires Rlim to be expressed from two alleles (1). However, disputing such model, results obtained by mouse genetics and tetraploid complementation assays has provided evidence that Rlim is dispensable for rXCI during female mouse embryogenesis (26). To further illuminate roles of the Rlim-Rex1 axis during rXCI *in vivo*, we investigated the dynamics of Rlim and Rex1 expression in post-implantation embryos with an emphasis on epiblast cells. We first interrogated embryo sections in uteri at E5.0, the timepoint when rXCI is initiated. Co-staining with antibodies against Rlim and Rex1, we found robust Rlim signals in Cdx2^+^ trophoblast tissues, whereas in epiblast cells, signals appeared notably weaker, consistent with published data (Fig. 4A) (26). Importantly and in contrast to pre-implantation stages, Rex1 was very low/nearly absent in WT embryos. In embryos lacking Rlim, Rex1 was robustly expressed in Cdx2^+^ extraembryonic tissues, indicating continued roles of Rlim in the turn-over of Rex1, but specifically in Cdx2-negative cells Rex1 levels appeared downregulated. To address the lineage of these cells, we performed co-staining with the epiblast cell marker Oct4. Indeed, our results reveal an Rlim-independent downregulation of Rex1 levels in Oct4^+^ epiblast cells (Fig. 4B), consistent with a functional uncoupling of the Rlim-Rex1 axis specifically in this cell type.

**Figure 5:**
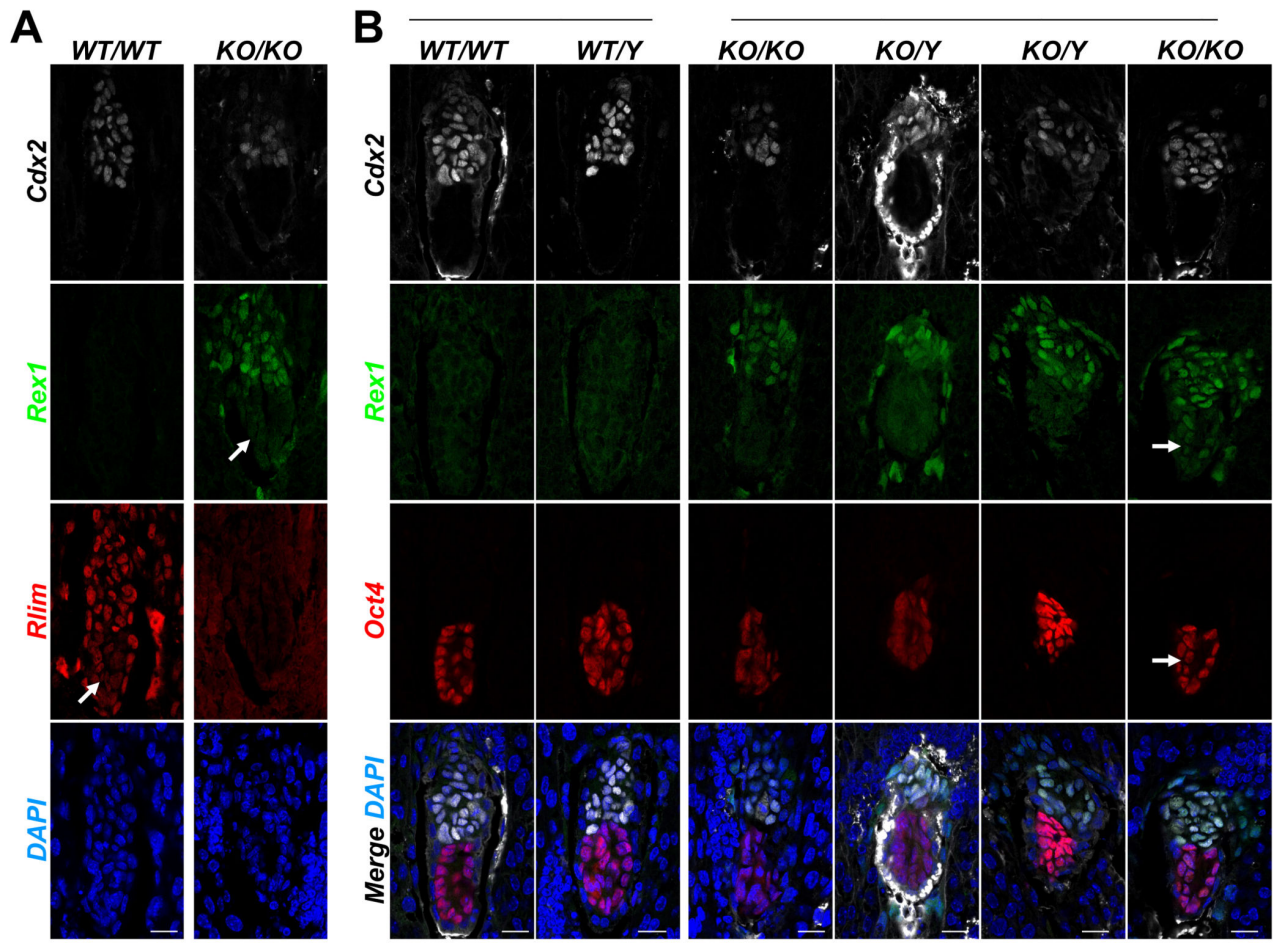
Low levels of Rex1 in epiblast cells of embryos lacking *Rlim* by E5.25. Sections of WT and Rlim KO embryos in uteri at post-implantation stage E5.25 were co-stained in parallel using indicated antibodies. Image recordings/processing of KO and control embryos were carried out in parallel using the same settings. **A)** Rlim=red; Rex1=green; Cdx2=white; DAPI=blue. Green and white arrows indicate exe and epiblast domains, respectively. **B)** Oct4=red; Rex1=green; Cdx2=white; DAPI=blue. White arrows indicate epiblast domains. Note low Rex1 immunoreactivity specifically in epiblast regions in embryos lacking *Rlim*. Arrow points at epiblast region in an embryo lacking Rlim displaying detectable levels of Rex1 (2 out of 10). Scale bars =50 μm.

To further investigate the kinetics of this uncoupling process as well as possible influences of sex, we interrogated E5.25 embryos. Again, Rlim immunoreactivity was robust in extraembryonic tissues, with reduced staining in epiblast cells (Fig. 5A, B). Independent of sex, Rex1 was very low/undetectable in WT embryos at this stage both in embryonic and extraembryonic tissues. Importantly, while extraembryonic tissues in the *Rlim* cKO continued to exhibit increased Rex1 levels, Rex1 was very low in Oct4^+^ cells of both male and female embryos (Fig. 5B). Indeed, out of ten embryos tested, we detected weak Rex1 staining in epiblast cells in only two embryos lacking Rlim at this stage (Fig. 5B). Quantification of Rex1 signals via ImageJ confirmed robust stabilization of Rex1 in Rlim KO trophoblast cells, but levels Oct4^+^ epiblast cells were only slightly above those of WT embryos (compare Figs. 5, S4). Examining whole embryos at post-implantation stages E5.5 and E6.5 revealed that low levels of Rex1 continue to be dependent on Rlim in trophoblast but not in epiblast tissues (Fig. S5). Combined, these data are consistent with a rapid differentiation-induced downregulation of Rex1 mRNA (8, 9), and first reveal the functional uncoupling of the Rlim-Rex1 axis specifically in epiblast cells, initiated before E5.0 in a sex-independent manner.

### Rlim-independent downregulation of Rex1 in epiblast cells allows for XCI

**Figure 6:**
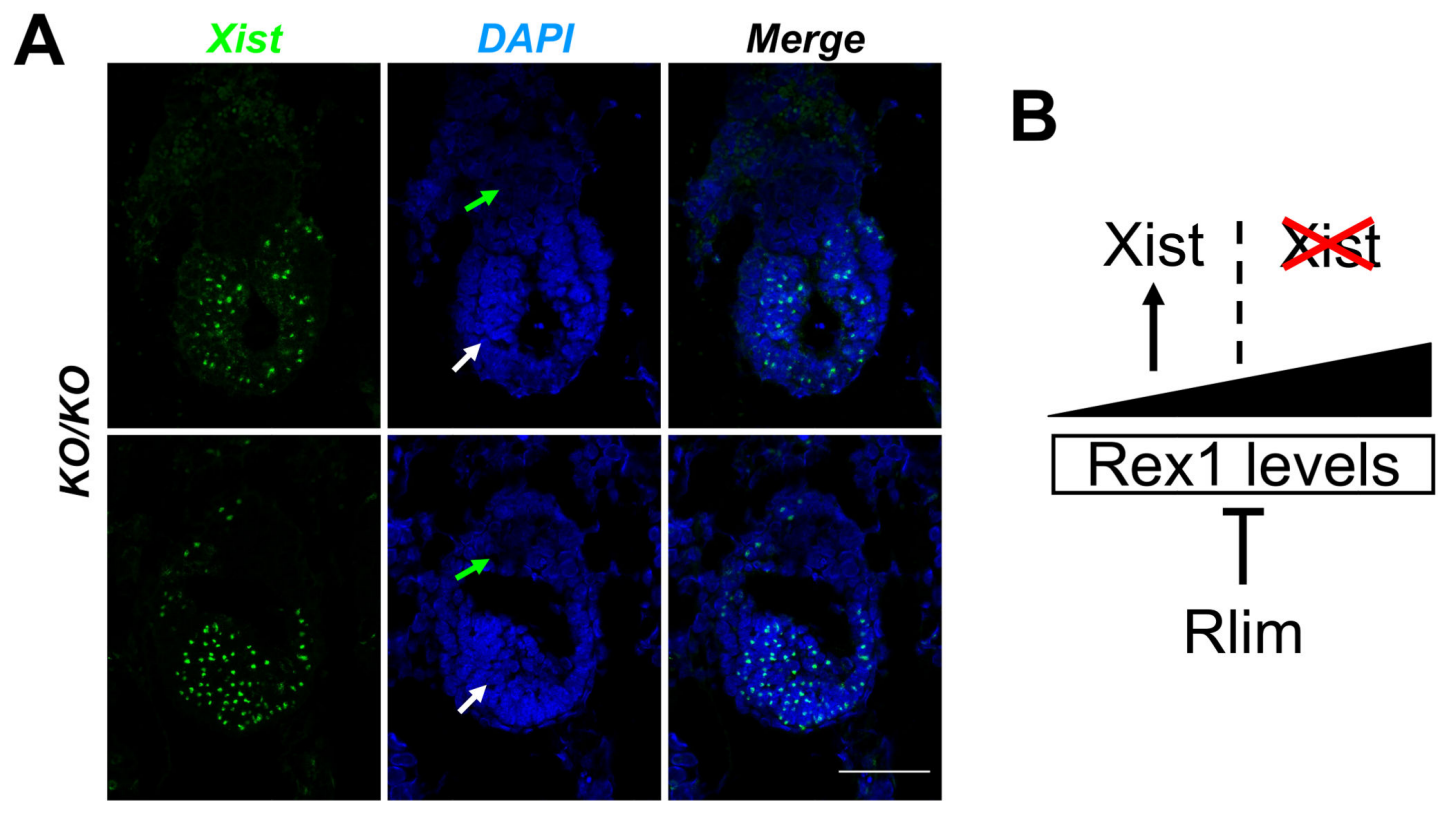
Rlim-independent activation of *Xist* in the embryonic epiblast. **A)** RNA FISH on sections of representative *Rlim* KO/KO female embryos in decidua at stages E7.5 using a *Xist* probe. Representative images depicting epiblast and bordering trophoblast embryonic regions. Green and white arrows indicate trophoblast and epiblast regions, respectively. Note presence of *Xist* paints in epiblast cells but very little in pTE-derived trophoblasts regions. Scale bars = 20μm. **B)** Model of how the Rlim-Rex1 axis regulates iXCI in female mice. Rlim activity keeps Rex1 below threshold levels required for activation of *Xist*. Note that it is the dose of Rex1 that is critical for activation of *Xist*, whether Rlim is present or not.

Because in female ESCs, high levels of Rex1 inhibits XCI (7), we addressed the question if the Rlim-independent downregulation of Rex1 in epiblast cells would allow for XCI. Thus, we investigated the state of XCI in female embryos lacking *Rlim* by RNA FISH, hybridizing with a probe specifically recognizing the lnc*Xist* that paints the inactive X chromosome, and by staining sections of embryos in decidua with antibodies against H3K27me3, a downstream marker of *Xist* (3). Because some female *Rlim* KO/KO embryos survive until E7.5 (5), which corresponds to the earliest stage when H3K27me3 foci induced by rXCI are detectable in the epiblast (26), we interrogated the state of XCI in female embryos at this stage. Indeed, all female embryos lacking Rlim (n=10) displayed *Xist* clouds (Fig. 6A; S6) or H3K27me3 foci in epiblast but not trophoblast tissues (Fig. S7). Combined, these results demonstrate that the Rlim-independent downregulation of Rex1 in epiblast cells allows for the induction of XCI, consistent with findings that *Rlim* is required for iXCI but not rXCI (26). These results combined with data showing *Xist* clouds transiently developing during iXCI in female embryos lacking Rlim at E2.5 when *Rlim* mRNA and protein levels are low (Fig. 3) (6, 38), provide a conceptual framework of how the Rlim-Rex1 axis regulates iXCI but not rXCI in mice. In this *in vivo* model the activation of *Xist* and XCI is dependent on levels of Rex1 below specific threshold levels, irrespective of whether Rlim is present (Fig. 6B).

## Discussion

In female embryos lacking *Rlim*, pre-implantation embryogenesis including the specification and development of initial trophoblast and epiblast tissues appears normal, but a highly penetrant lethal embryonic phenotype precipitates upon implantation^5,6^. While general post-implantation development is severely stalled, the epiblast cell lineage does not appear to display major defects, as female epiblast tissues lacking *Rlim* undergo lumenogenesis (Fig. 1), downregulate Sox2 expression (Fig. 2), develop *Xist* clouds and H3K27me3 foci (Figs. 6; S4), and continue to express Fgf4 (Fig. 2). Moreover, differentiation of mTE-derived parietal TGCs lacking Rlim occurs in a similar fashion to control animals (Figs. 1; Suppl. Fig. 1). Thus, the main phenotype in *Rlim* KO female embryos appears mostly restricted to pTE progenitor cells and derived structures. Indeed, pTEs in female embryos lacking X dosage compensation prematurely differentiate without self-maintenance, unable to develop the exe structure. The finding that pTEs cells of some embryos lacking *Rlim* differentiate into Mash2^+^ cells but not those in other embryos (Fig. 2), indicates inconsistent differentiation into various cell fates as trophoblast cell lineages other than Mash2^+^ spongiotrophoblasts exist that do not express Mash2, including the labyrinthine trophoblast lineage (39). The loss of lineage-defining factors including Cdx2, Eomes and Esrrb (11, 20, 24) in pTEs likely contributes to such cell fate confusion. In this context, considering that reciprocal repression between Oct4 and Cdx2 is important for initial trophoblast specification^21^, the downregulation of Cdx2 in female pTEs lacking X dosage compensation (Figs. 3; Fig. S2) may allow for the reversion of Oct4 expression in some pTE cells (Fig. 5C). However, even though epiblast cells generally display low migration activity, we cannot exclude that the presence of Oct4^+^ cells in trophoblast regions might be caused by migration of cells from epiblast regions. Because the Rlim-Rex1 axis selectively regulates the process of iXCI, the developmental phenotypes observed in females lacking Rlim are in strong support of a cell autonomous requirement of X dosage compensation in pTEs. Such scenario is consistent with the finding that pTEs are also sensitive to *Xist* deletion (25), in contrast to epiblast-derived tissues (40). What makes the pTE progenitor cell lineage particularly vulnerable to X dosage compensation defects? In ESCs, the X dosage influences the pluripotency network via MAPK signaling, and a double dose of X-linked MAPK regulator genes such as Dusp9 and Klh113 represses MAPK signaling, thereby activating pluripotency factor expression (41–44). Because MAPK signaling also regulates cell proliferation (14, 45), it is plausible that high levels of X-linked MAPK regulators participate in causing the phenotypes observed in *Rlim* KO pTE progenitors, including inhibition of Fgf4 signaling, cell proliferation as well as cell fate confusion (Figs. 1, 2).

Concerning the functions of the Rlim-Rex1 axis in female pre-implantation embryos, iXCI gradually silences Xp-linked genes until X dosage compensation is reached at late blastocyst stages, including in trophoblast cells (6). The overall pattern of Rlim and Rex1 expression during pre-implantation development in WT controls, combined with elevated Rex1 levels in *Rlim* KO animals fully support crucial functions of this axis in controlling iXCI, with Rlim targeting Rex1 for proteasomal degradation (7, 10). It is important to point out that Rex1 protein is low but detectable throughout pre-implantation development in animals WT for *Rlim* (Fig. 3), indicating that much of the process of iXCI occurs in the presence of low levels of Rex1. Indeed, signs of *Xist* activation and cloud formation at early stages of mouse pre-implantation development have been reported in females lacking Rlim by E2.5 (6), when *Rex1* mRNA levels are low (38). Consistent with this, expression data from animals at E2.5 indicate low levels of Rex1 protein, with levels only slightly increasing upon deletion of *Rlim* (Fig. 3). In contrast, the requirement for Rlim activity to maintain Rex1 at low levels appears most critical at E3.5, when *Rex1* mRNA expression is high (6, 38). These results are not only fully consistent with whole embryo RNAseq data on *Xist* expression and X-silencing profiles, but also the collapse of *Xist* clouds in female embryos lacking *Rlim* at blastocyst stages (6), indicating that Rex1 levels in these embryos have reached threshold levels that inhibit *Xist* expression.

With respect to roles of the Rlim-Rex1 axis during rXCI in post-implantation embryos, our results show that the Rlim-Rex1 axis continues to be active in trophoblast tissues. However, in epiblast tissues, this functional interaction is severed by an abrupt Rlim-independent downregulation of Rex1 at the transcriptional level (8, 9), likely initiated at implantation (Fig. 4) and mostly achieved by E5.25, just 6h after initiation of rXCI (Figs. 5; S4). We show further that suppression of Rex1 levels occurs within a time frame when cells are competent for initiation of *Xist* transcription, allowing rXCI to occur (Figs. 6; S7). These results not only provide a molecular explanation of why Rlim is not required for rXCI in mice (26), but also a mechanism for the Rlim-Rex1 function specifically in extraembryonic tissues. Combined, these results indicate that activation of *Xist* in female embryos is dependent on doses of Rex1 below threshold levels during both iXCI and rXCI (Fig. 6C), irrespective of the *Rlim* status.

Regarding rXCI, our results identify important differences between epiblast cells and ESCs with respect to the Rlim-Rex1 axis, as ESCs represent cells at pre-implantation stages when the Rlim-Rex1 axis is active. Thus, varying Rex1 levels in different ESC lines may account for the reported varying degree of Rlim requirement for rXCI *in vitro* (35, 46). The findings of *Xist* activation in female embryos lacking Rlim appear incompatible with the current ESC-derived model of XCI initiation that requires high Rlim doses expressed from two alleles (1), which needs to be re-evaluated.

In summary, our data show a decisive role of the functional dynamics between Rlim and Rex1 in the control of iXCI but exclude essential functions for rXCI in female mice. Moreover, our results reveal that cell autonomous X dosage compensation via the Rlim-Rex1 axis is required to maintain pTE progenitor cell identity, crucial for successful mouse development.

## Material and Methods

### Antibodies

Concerning the generation and characterisation of the REX1 antibody, a sheep anti-mouse REX1 (1-288) antibody was raised by MRC PPU Reagents & Services (MRC PPU R&S code DA136). GST-tagged mouse REX1 (1-288) was used as the immunogen and the serum purified against MBP-tagged REX1 (1-288) to minimise purification of GST antibodies. Antibody specificity was determined by immunofluorescence and immunoblotting. For immunoblotting, *Rlim* ^+/y^, *Rlim* ^-/y^ or *Rlim* ^-/y^ ; *Zfp42* ^-/-^ mESCs (47, 48) were cultured on gelatin and lysed in 20 mM tris-HCl (pH 7.4), 150 mM NaCl, 1 mM EDTA, 1% (v/v) NP-40, 0.5% (w/v) sodium deoxycholate, 10 mM β-glycerophosphate, 10 mM sodium pyrophosphate, 1 mM NaF, 2 mM Na_3_VO_4_, and cOmplete Protease Inhibitor Cocktail Tablets (0.1 U/ml; Roche) and protein extracts resolved by SDS-PAGE prior to transfer. REX1 antibody DA136 (3^rd^ bleed) was diluted at 1:1000 and visualized using Donkey anti-Sheep HRP secondary antibody (Thermo Fisher Scientific). Immunoblots were imaged using enhanced chemiluminescence on a BioRad ChemiDoc imager (BioRad). For immunostaining, *Rlim* ^+/y^, *Rlim* ^-/y^ or *Rlim* ^-/y^; *Zfp42* ^-/-^ mESCs (47, 48) were cultured on gelatin coated coverslips, fixed with 4% PFA in PBS for 20 min, permeabilized in 0.5% Triton X-100 PBS 5 min and blocked with 1% Fish gelatin PBS 30 min. REX1 antibody DA136 (3^rd^ bleed) was diluted at 1:1000 in blocking solution and coverslips were incubated for 2 h. Alexa Fluor 488 Donkey anti-Sheep IgG (Thermo Fisher Scientific) was used as secondary antibody at 1:500 in blocking solution and incubated for 1 h. DNA stain was performed with Hoechst (1:10,000 in PBS) for 5 min. Coverslips were mounted using Fluorsave reagent and imaged using a Zeiss 710 confocal microscope with Zen software (Zeiss). The N-Rlim antibody has previously been described (4, 35). Other commercial primary antibodies were Cdx2 (Biogenex MU392A-UC), pH3 (Millipore NG1740993), Oct4 (Abcam, 200834), E-cadherin (BD biosciences 610181), Eomes (Abcam 23345), H3K27me3 (Millipore Sigma 07-449), pErk1/2 (Cell Signaling #9102), Fgfr2 (Abcam 10648), Casp3 (Cell Signaling #9661), Sox2 (Abcam 97959). Secondary antibodies were purchased from Thermo Fisher Scientific and include Alexa Fluor® 488 Donkey Anti-Rabbit IgG (A21206), Alexa Fluor® 488 Goat Anti-mouse IgG (A11029), Alexa Fluor® 568 Goat Anti-Rabbit IgG (A-11011), Alexa Fluor® 568 Goat Anti-rat IgG (A-11077), and Alexa Fluor® 568 Goat Anti-mouse IgG (A-11004), Alexa Fluor® 488 Donkey anti-sheep IgG, (A-11015), Alexa Fluor® 546 Donkey anti-Rabbit IgG (A-10040), Alexa Fluor® 647 Donkey anti-Mouse IgG, (A-31571).

### Immunostaining of mouse embryos

Concerning the determination of embryo stages, noon of the day when mating plugs were observed was considered as embryonic day 0.5 of development (E0.5). Embryo dissections, preparation and culturing of blastocyst outgrowths, and immunostaining of whole mouse embryos, embryonic sections and blastocyst outgrowths as well as sex determination via PCR was performed as previously described (5, 26). The staining of KO and control embryos were carried out in parallel at each stage, with image recordings and processing performed using the same settings. Statistical analyses were performed using Student’s t test. The quantification of Rex1 signals on immunostainings were performed using ImageJ, normalized against DAPI on at least 2 biological replicates.

### Wholemount In situ hybridizations of mouse embryos

In situ hybridizations on dissected embryos were performed as previously described (Henrique et al., 1995; Yoon et al., 2013), including embryo dissections and the preparation and hybridization of probes. Briefly, embryos were dissected at stages E5.5 and E6.5. Noon of the day when mating plugs were observed was considered as embryonic day E0.5. Using forceps, dissections were performed in the dissection medium (DMEM (Gibco) containing 20 mM Hepes, 10% FCS, 100 U/ml penicillin and 100 μg/ml Streptomycin). Established plasmids have been described for making antisense probes recognizing *Wnt-7b* (Yoon et al., 2013), *Mash2, Esrrb* and *Fgf4* (29).

### RNA-FISH

RNA-FISH on sections of mouse embryos within placentae were carried out using a kit (Molecular Instruments) containing an HCR™ Probe (Xist, Lot: RTA603), HCR amplifier (B1, Fluorophore Alexa488) and hybridization, wash, and amplification buffers. Sequences for HCR amplifiers B1, B2, B3, B4, and B5 are given in (Choi et al., 2014). In situ hybridization was performed after sectioning FFPE samples following Molecular Instruments protocol (molecularinstruments.org).

## Supplementary Material

Supplemental figures

## Data Availability

All study data are included in the article and/or SI Appendix. Resources generated in this study are available upon request.

## Significance

The ubiquitin ligase Rlim in conjunction with its substrate protein Rex1 are crucial regulators of X chromosome inactivation (XCI), the transcriptional silencing of one X chromosome, required for successful female mouse embryogenesis. However, their contribution toward this process in specific mouse tissues has been controversial. Here, we demonstrate a crucial functional interaction of the Rlim-Rex1 axis during pre-implantation stages for the development of the extraembryonic ectoderm in females. We show further that in contrast to extraembryonic tissues, the functionality of this axis is severed specifically in epiblast cells upon implantation. Thus, a functional Rlim-Rex1 axis is required for X dosage compensation specifically in extraembryonic tissues of female mice.
