# Supplementary material for: Roles of the Rlim-Rex1 axis during X chromosome inactivation in mice
Source: Proc Natl Acad Sci U S A. Author manuscript; Available in PMC 2024 Mar 6. (PMC10756295; doi:10.1073/pnas.2313200120)

SUPPLEMENTAL FIGURE LEGENDS

Figure S1: Collapse of a Cdx2^{high} cell pool and defective pTE function in female *Rlim* KO/KO blastocyst outgrowths. A) Efficient generation of mouse offspring lacking *Rlim*. Parental and offspring genotypes are indicated. **B)** Male/female *Rlim*KO blastocysts cultured *in vitro*. While similar numbers of Cdx2^{high} cells are detected in male and female blastocyst outgrowths cultured for 1 day (**d1)**, by **3d** females start losing Cdx2^{high} trophoblast cells (mostly lost by 4d), with similar numbers of pH3^{+} mitotic cells. At **5d** (brightfield) males but not females have formed large, presumably pTE-derived structures (green arrow). White arrows indicate disorganized epiblast cells. n>10, each. Genotyping after image recording. **C)** Quantification of outgrowths exhibiting inhibited pTE-derived cell growths at 5d.

Figure S2: High specificity of Rex1 antibodies. Rex1 antibodies generated in sheep (3rd bleed) were tested using male ESC models including ESCs WT for *Rlim* and Rex1 (*Rlim* +/y; *Zfp42* +/+), ESCs lacking *Rlim* (*Rlim* -/y; *Zfp42* +/+), and ESCs lacking both (*Rlim* -/Y; *Zfp42* -/-) via **A)** Western blotting, and **B)** immunostaining.

Figure S3: Increased Rex1 protein levels in E3.5 pre-implantation embryos lacking Rlim. A) Rlim- and sex-independent levels of Zfp42 (Rex1) mRNA in E3.5 embryos as determined by analysis of a previously published single embryo RNA-seq dataset (6). Differences in mRNA levels between genotypes are not significant. **B)** IHC on mouse embryos at E3.5 showing DAPI (blue), Cdx2 (white) and Rex1 (green) staining. Representative images are shown. Sex was determined after image recordings. Scale bar = 25µm. **C)** Quantification of Rex1 signals in ICM

and trophoblast cells of E3.5 embryos using ImageJ. Relative values are shown in relation to ICM cells in WT/Y (=1). Note that increased relative Rex1 levels both in ICM and trophoblast cell types depend on *Rlim* status but not on sex. n>55 cells, each.

Figure S4: Rlim-independent downregulation of Rex1 protein in epiblast cells of post-implantation embryos. Quantification of Rex1 signals in epiblast and trophoblast cells of E5.25 embryos using ImageJ. Relative values are shown in relation to WT/Y epiblast levels (=1), which are very low. While Rex1 protein levels are only slightly increased in male and female KO epiblast cells, they show robust stabilization both in male and female trophoblast cells. n>30 cells, each.

Figure S5: Rlim-independent downregulation of Rex1 in epiblast cells. WT and *Rlim* KO embryos at post-implantation stages E5.5 and E6.5 were co-stained in parallel using indicated antibodies. Image recordings/processing of KO and control embryos were carried out using the same settings. Rlim=red; Rex1=green; E-cad=white. DAPI=blue. Green and white arrows indicate exe and epiblast domains, respectively. Note increased Rex1 immunoreactivity specifically in trophoblast but not epiblast regions in embryos lacking *Rlim*. Scale bars =50 μm.

Figure S6: Rlim-independent activation of *Xist* in the embryonic epiblast. A) RNA FISH control experiments on sections of WT embryos in decidua at stage E7.5 using a *Xist* probe. Green and white arrows indicate trophoblast and epiblast regions, respectively. Note presence of *Xist* paints in cells of both tissues in female embryos. Scale bar = 100μm.

Figure S7: HeK27me3 foci form in epiblast tissues of females lacking *Rlim*. Sections of WT/Y male, and WT/WT and *Rlim* KO/KO female embryos in decidua at stages E7.5 were stained with H3K27me3 antibodies. Shown are representative images depicting epiblast and bordering trophoblast embryonic regions. Boxed area is shown in higher magnification. Note presence of small H3K27me3 foci in female KO/KO epiblast but not trophoblast tissues. Larger spots are likely caused by unspecific staining of blood accumulations. Scale bars = 50µm.

Wang et al., Suppl. Fig. 1

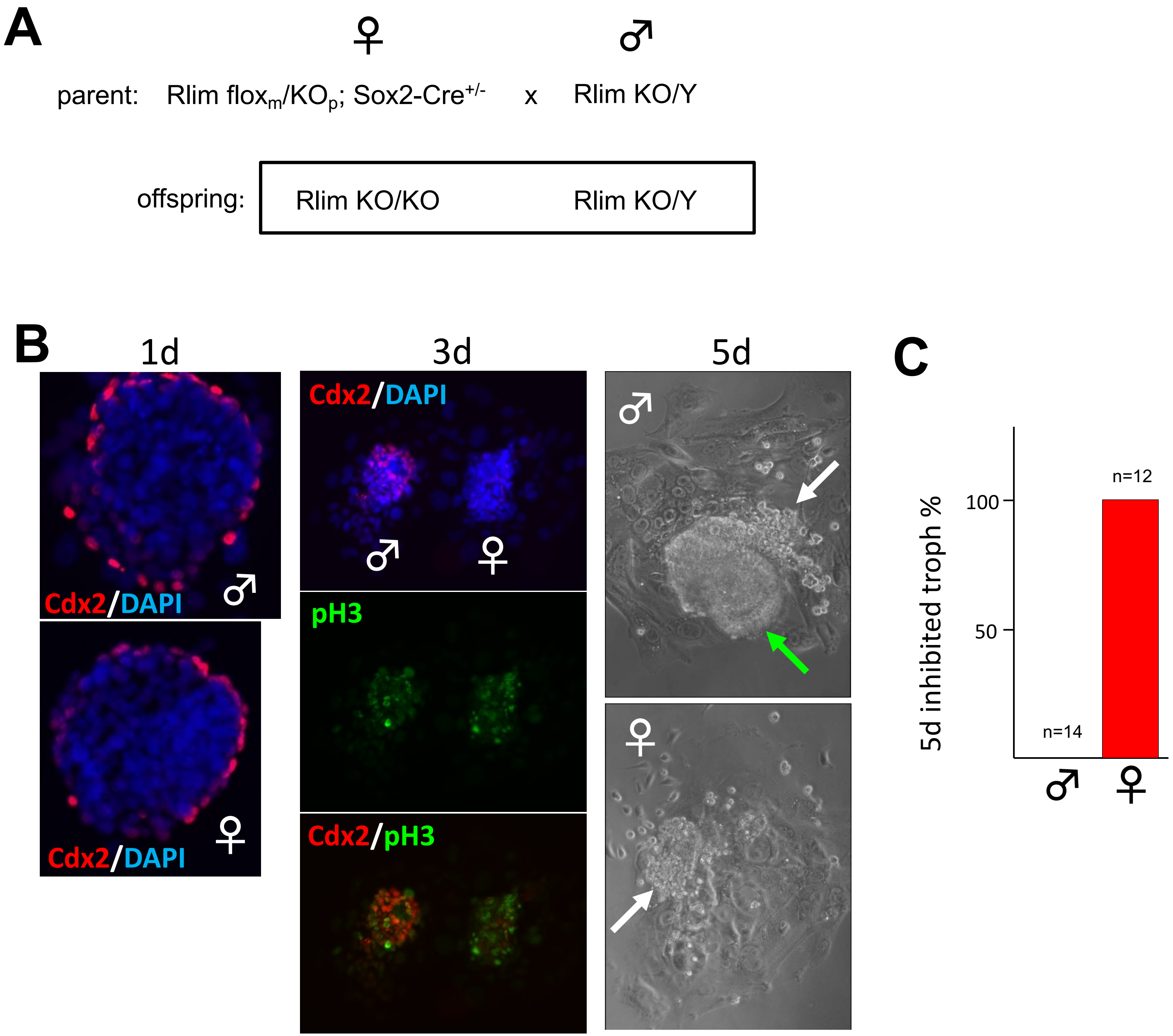

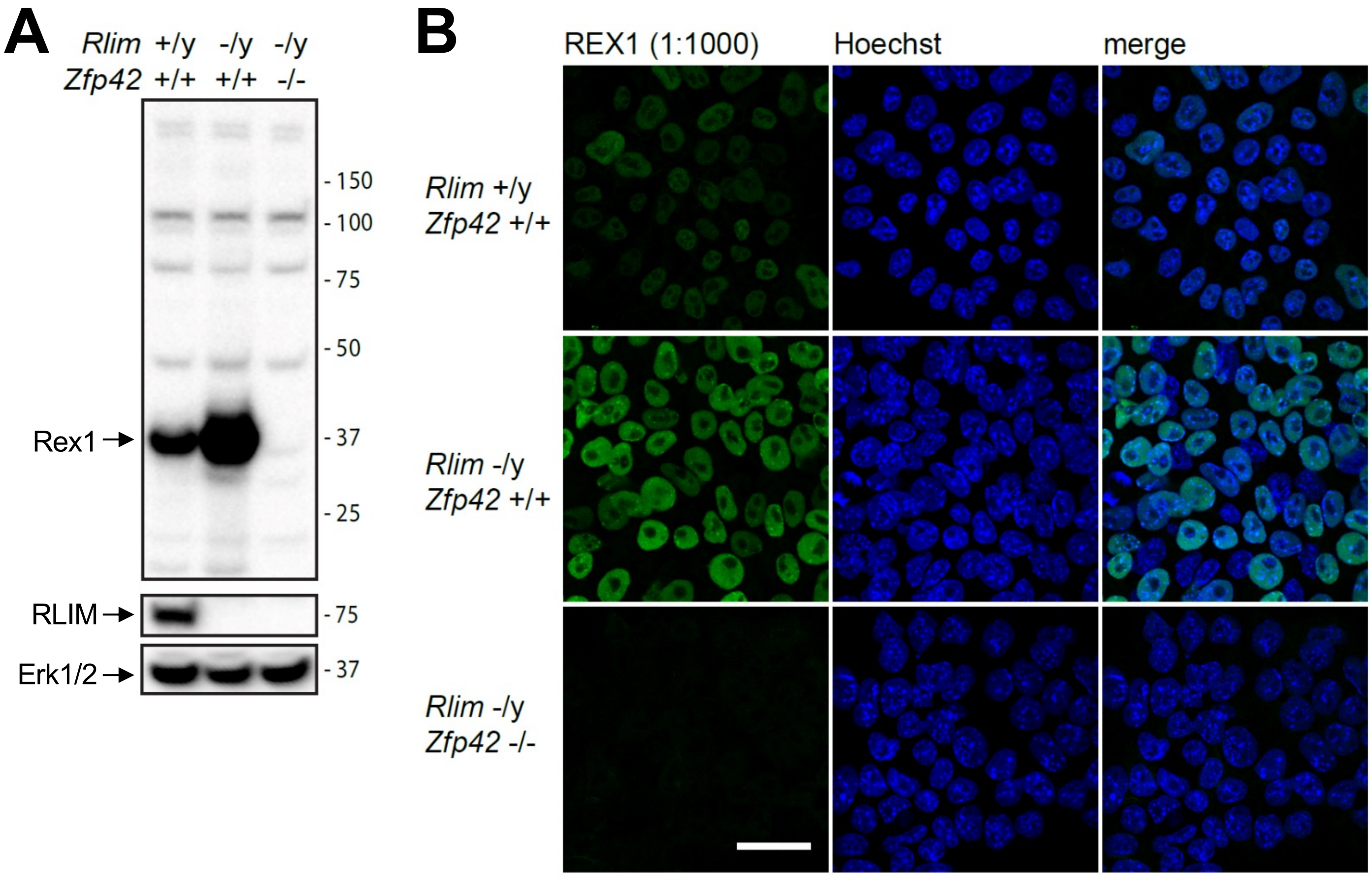
A
Rlim +/y -/y -/y
Zfp42 +/+ +/+ -/-
- 150
- 100
- 75
- 50
- 37
- 25
Rex1
RLIM
- 75
Erk1/2
- 37
B
REX1 (1:1000)
Hoechst
merge
Rlim +/y
Zfp42 +/+
Rlim -/y
Zfp42 +/+
Rlim -/y
Zfp42 -/-

Wang et al., Suppl. Fig. 3

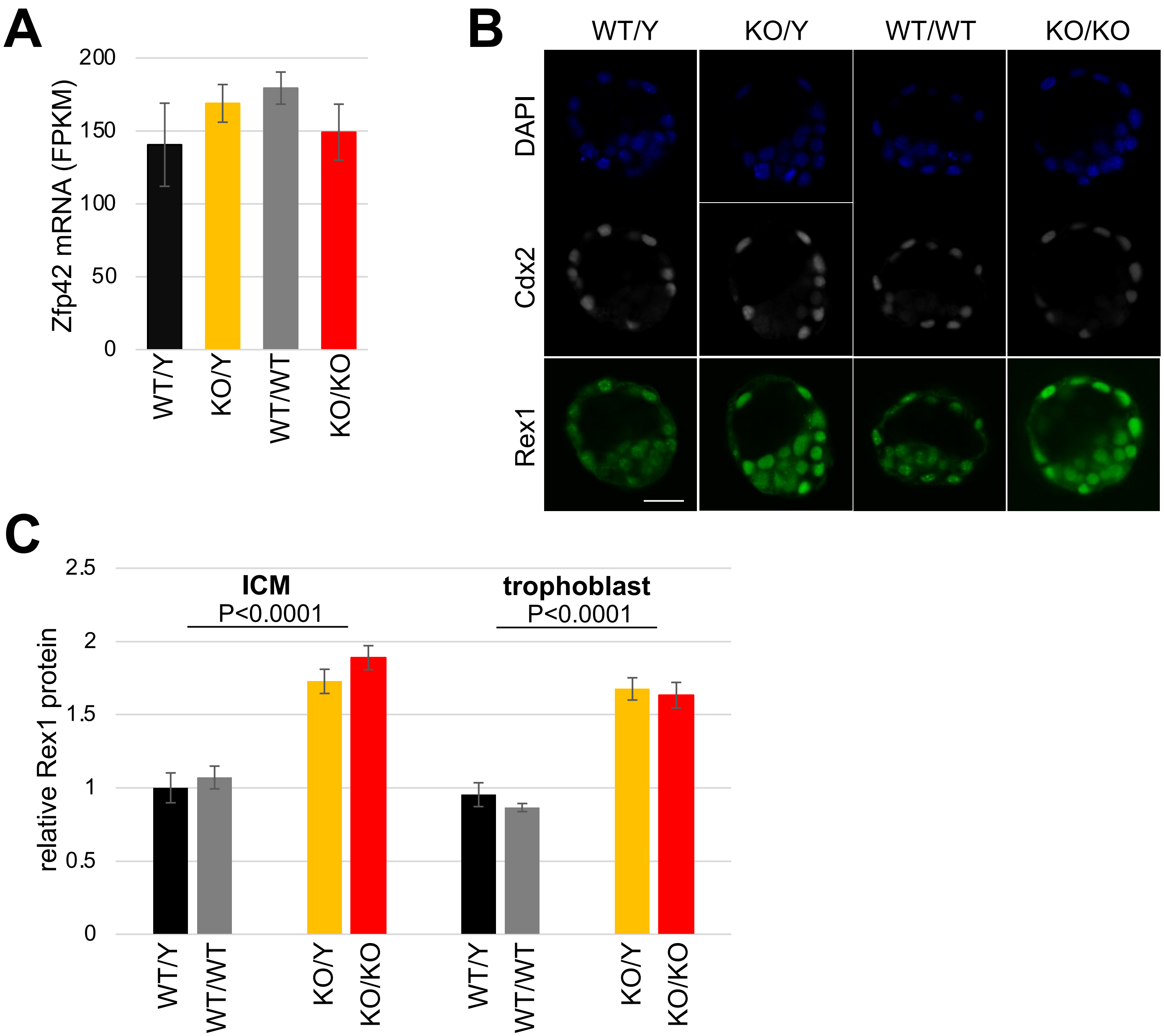

Wang et al., Suppl. Fig. 4

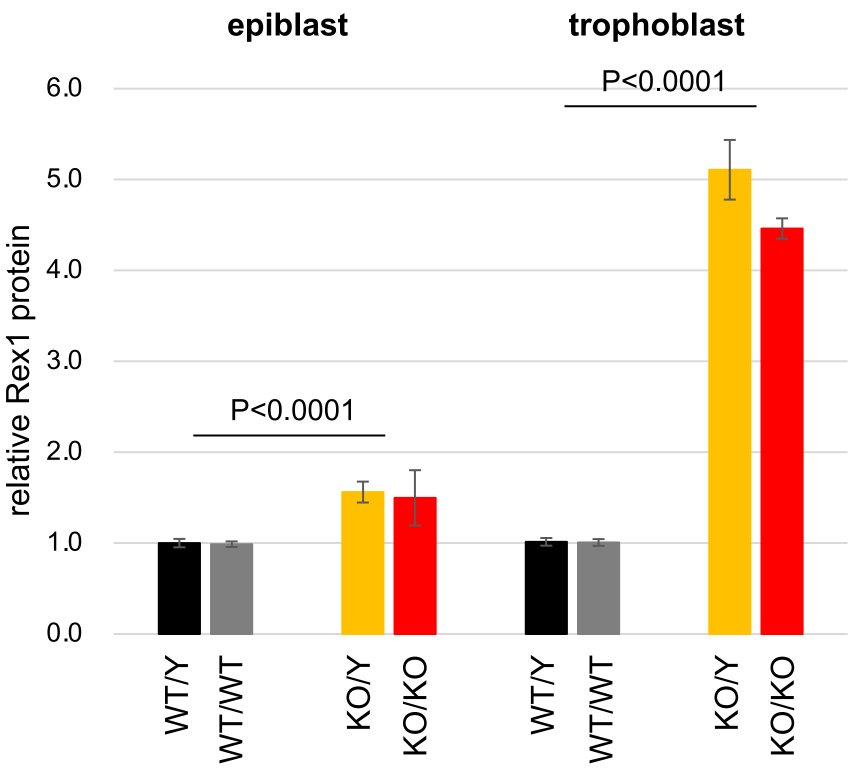

Wang et al., Suppl. Fig. 5

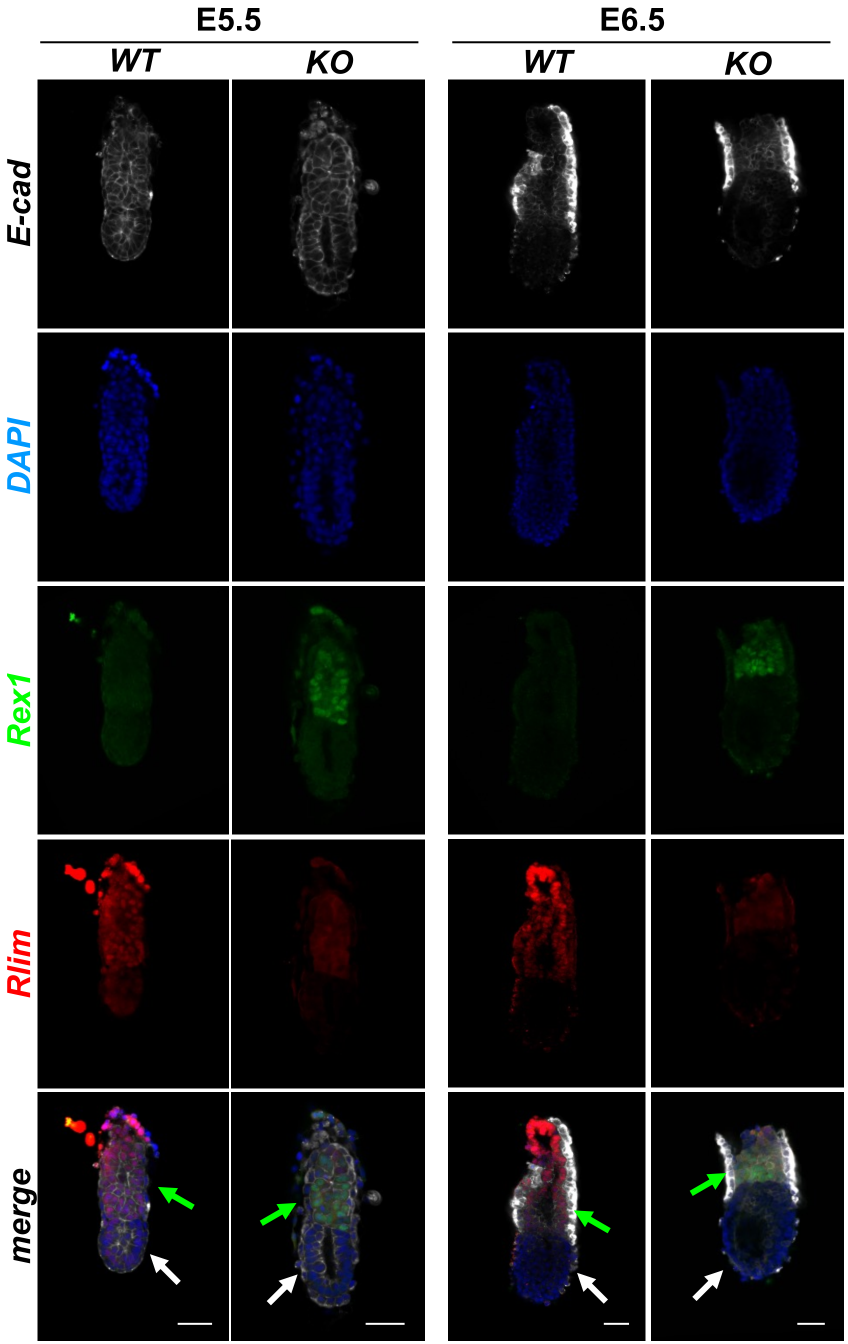

Wang et al., Suppl. Fig. 6

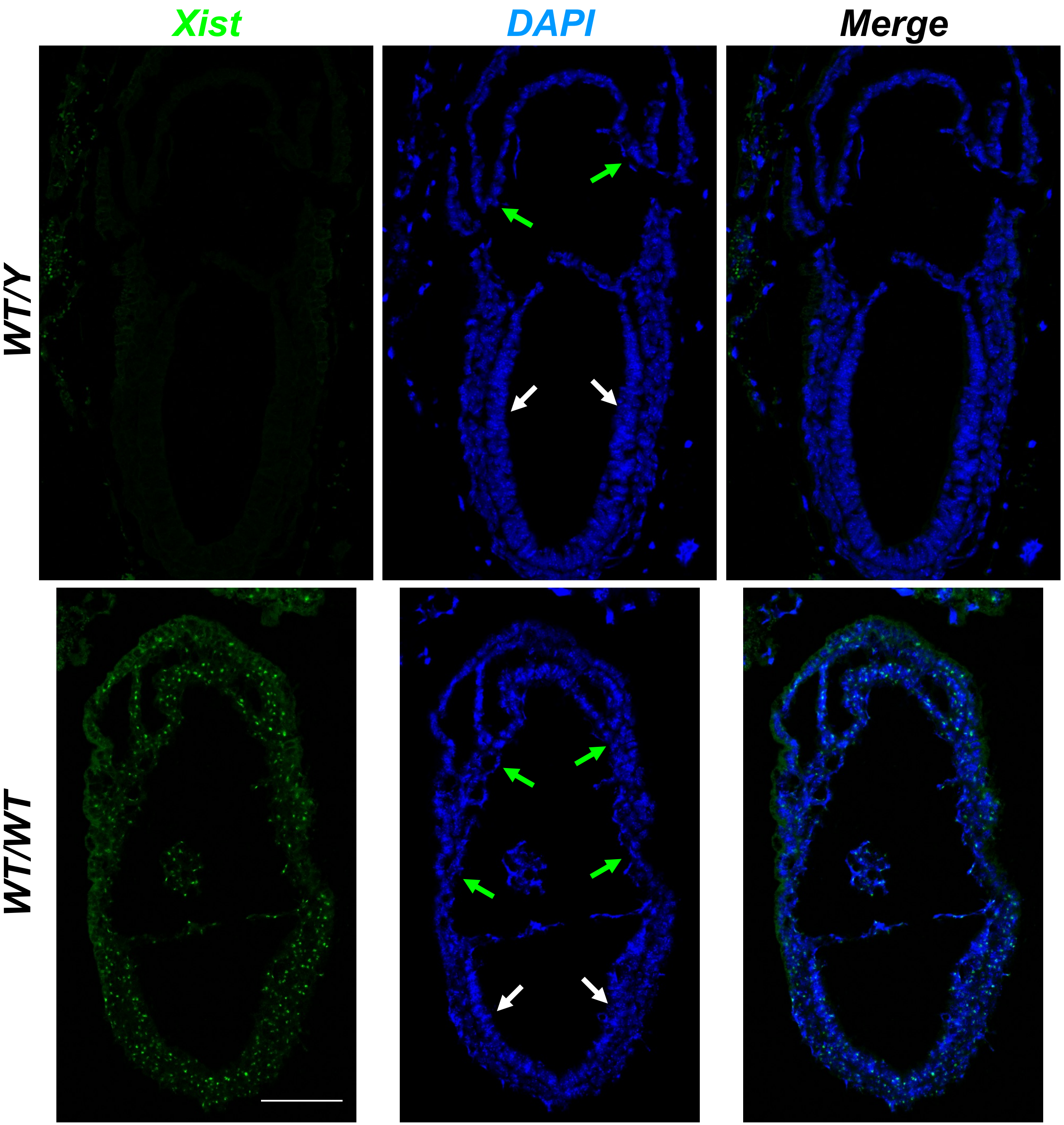

Wang et al., Suppl. Fig. 7

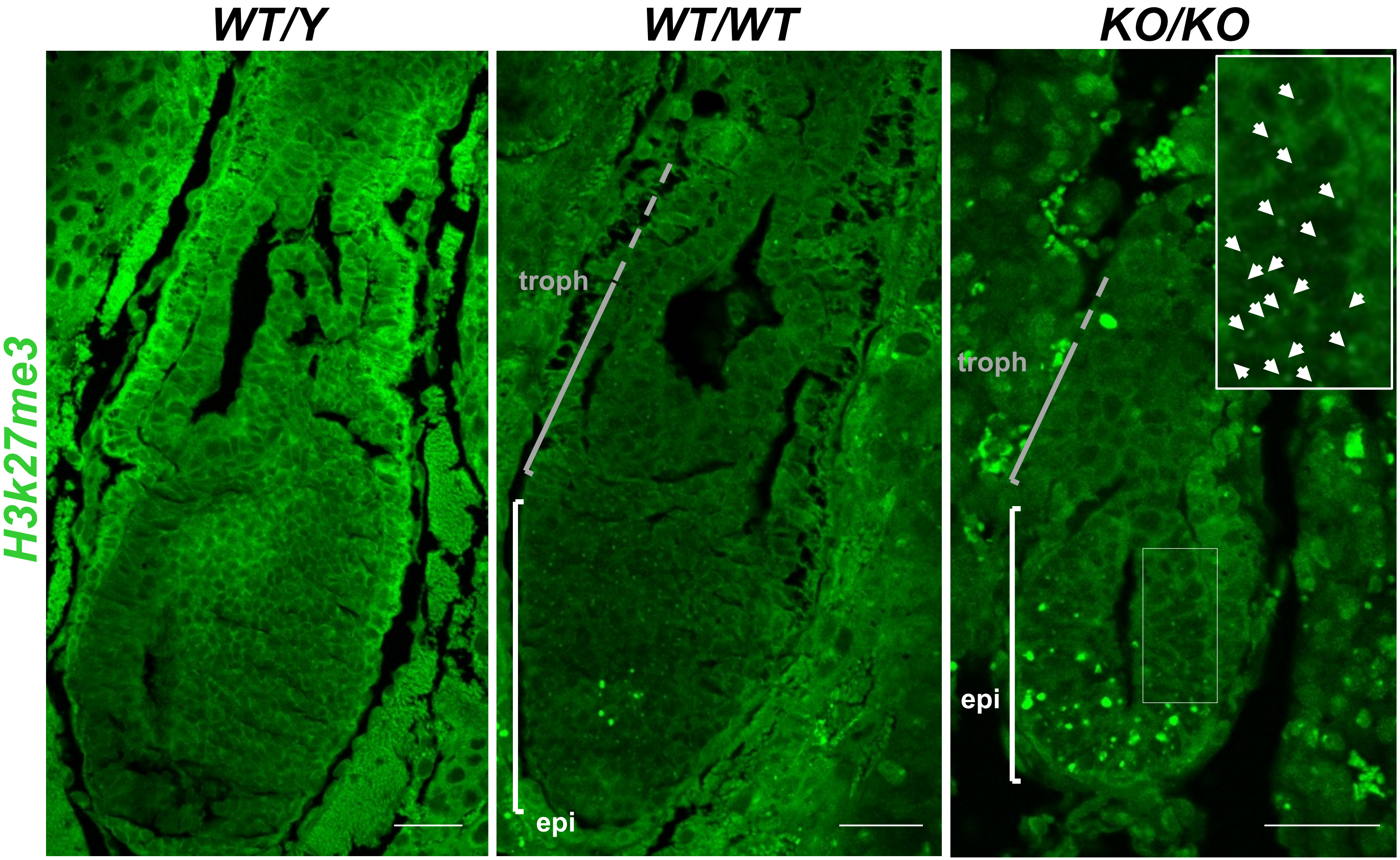

Supplement: Supplemental figures [file EMS194350-supplement-Supplemental_figures.pdf]
